# Fracture of distal humerus: MIPO technique with visualization of the radial nerve

**DOI:** 10.1590/1413-78522014220601003

**Published:** 2014

**Authors:** Daniel Romano Zogbi, Alberto Maranon Terrivel, Guilherme Grisi Mouraria, Maurício Leal Dias Mongon, Fernando Kenji Kikuta, Américo Zoppi Filho

**Affiliations:** 1. Universidade Estadual de Campinas, Department of Orthopedics and Traumatology, Campinas, SP, Brazil, Department of Orthopedics and Traumatology, Universidade Estadual de Campinas (Unicamp), Campinas, SP, Brazil

**Keywords:** Humeral fractures, Diaphyses, Radial nerve, Peripheral nerve injuries

## Abstract

**OBJECTIVES:**

To evaluate the outcomes in patients treated for humerus distal third fractures with MIPO technique and visualization of the radial nerve by an accessory approach, in those without radial palsy before surgery.

**METHODS:**

The patients were treated with MIPO technique. The visualization and isolation of the radial nerve was done by an approach between the brachialis and the brachiorradialis, with an oblique incision, in the lateral side of the arm. MEPS was used to evaluate the elbow function.

**RESULTS:**

Seven patients were evaluated with a mean age of 29.8 years old. The average follow up was 29.85 months. The radial neuropraxis after surgery occurred in three patients. The sensorial recovery occurred after 3.16 months on average and also of the motor function, after 5.33 months on average, in all patients. We achieved fracture consolidation in all patients (M=4.22 months). The averages for flexion-extension and prono-supination were 112.85° and 145°, respectively. The MEPS average score was 86.42. There was no case of infection.

**CONCLUSION:**

This approach allowed excluding a radial nerve interposition on site of the fracture and/or under the plate, showing a high level of consolidation of the fracture and a good evolution of the range of movement of the elbow.** Level of Evidence IV, Case Series**

## INTRODUCTION

The humeral shaft fractures are common and account for approximately 3-5% of the occurrence of all types of fracture. They occur in a bimodal way, between 21 and 30 years old, and secondly in older patients, between 60 and 80 years old. However, the specific fracture of the distal third is rarer, accounting for about 0.6 to 1%.^1^^,^ 2

The approach of this type of fracture by using the minimally invasive osteosynthesis technique (MIPO) was well described by several authors, showing good results and configuring it as a reproducible option treatment.^3^^-^ 9

In fractures of the distal third of the humeral shaft there is a close proximity of the radial nerve and the fracture, since in this location occurs the passage of the nerve to the anterior compartment, through the lateral intermuscular septum. At this point, the nerve has lower mobility and is closer to the bone. Thus, there is a greater concern for iatrogenic nerve injury after reduction of the fracture.^10^^-^ 12

Technically, the surgical treatment of fractures of the distal humerus by MIPO technique implies a closeness between the plate and the nerve.^13^ Thus, due to the high risk of interposition in the fracture and the close proximity of the nerve to the plate, a modification of the original technique was made, associating to it an accessory pathway for direct observation of this structure and exclusion of a possible radial nerve interposition in the fracture site and/or in the plate.

The study aimed to evaluate the clinical outcomes in patients who underwent osteosynthesis of fractures of the distal humerus through the MIPO technique and direct visualization of the radial nerve through an accessory pathway in patients who had no neurological deficit pre-operatively.

## MATERIALS AND METHODS

A retrospective study of a case series was conducted using data collected from medical records of 15 patients with closed fracture of the distal third of the humeral shaft, and treated with the MIPO technique treated at the Orthopedics ward.

The indication for surgical approach was the failure to obtain and maintain adequate closed reduction, from the following radiographic parameters: shortening greater than 3 cm, rotation greater than 30° and angle greater than 20°.^2^

**Figure 1:**
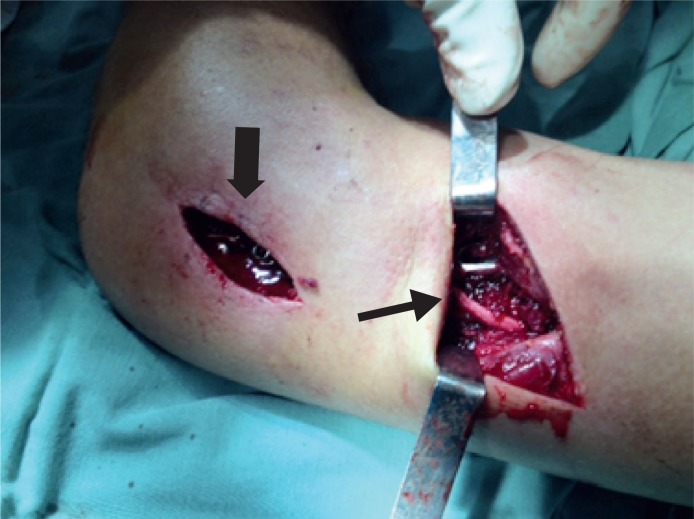
Kocher distal access way (thick arrow) and accessory access way with radial nerve isolated (thin arrow). Note plate below nerve.

The surgical technique used was a proximal approach between the biceps tendon medially and the deltoid tendon (deltopectoral). The distal access was performed as described by Kocher.^14 ^Since the display and isolation of the radial nerve were obtained with an approach between the arm and the brachioradialis, by an oblique incision of approximately 5-8 cm at the junction of the middle and distal thirds of the lateral side of the arm. (Figure 1) After making the incisions, a DCP (Dynamic Compression Plate) plate with 12 holes was placed percutaneously by the distal approach and fixed with two distal and two proximal screws. Bone healing was assessed by simple X-rays of the humerus and elbow, using digital technology with the Synapse(r) software.

The range of motion of the elbow was evaluated by values ​​in degrees recorded in medical records, previously measured with a simple goniometer. For the functional evaluation of the elbow, we used the Mayo Elbow Performance Score (MEPS).^15^

Neuropraxia of the radial nerve was assessed by levels of paresthesia and degree of strength (0-5) according to the scale of the British Medical Research Council. We also evaluate the recovery time of the neurological injury. The study was approved by the local Ethics Committee.

## RESULTS

Between 2008 e 2014 15 patients with fracture of the distal third of the humeral shaft were surgically treated. Among them, one patient was excluded for loss of outpatient follow-up, four were excluded because they were not submitted to the accessory pathway for direct visualization of the radial nerve and three were excluded because of the realization of preoperative neurological injury when data was collected from the medical records.

Of the seven patients included in this study, five are male and two females, with a mean age of 29.8 years old (St.Dev. ±11.88). Regarding laterality, six patients (85%) were injured on the left humerus and one (15%) on the right side. The mean follow-up was 29.85 months (St.Dev. ±21.94). 

The postoperative neuropraxia occurred in three patients. There was radial nerve sensory recovery in all patients, on average 3.16 months (St.Dev. ± 3.32) after the procedure. The motor recovery of the radial nerve also occurred in all patients, but after an average of 5.33 months (St.Dev. ±1.52). (Table 1)

**Table 1:** Postoperative assessment of the radial nerve.

Patient	Postoperative neurological deficit	Time for motor recovery (months)	Post recovery degree of muscle strength	Time for sensitive recovery (months)
1	No	-	-	-
2	No	-	-	-
3	No	-	-	-
4	No	-	-	-
5	Yes	5	5	1.5
6	Yes	4	4	1
7	Yes	7	4	7
Mean/St. Dev		5.33 (±1.52)	-	3.17 (±3.32)

**Figure 2:**
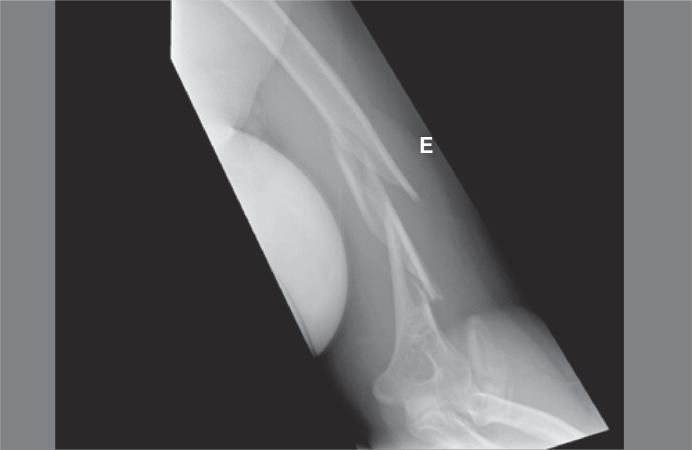
Preoperative X-Ray (AP) of patient with fracture of the distal third of the humeral shaft.

**Figure 3:**
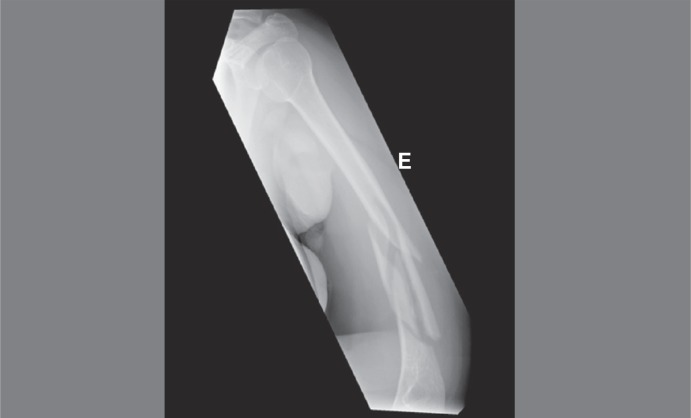
Preoperative X-Ray (Profile) of patient with fracture of the distal third of the humeral shaft

**Figure 4:**
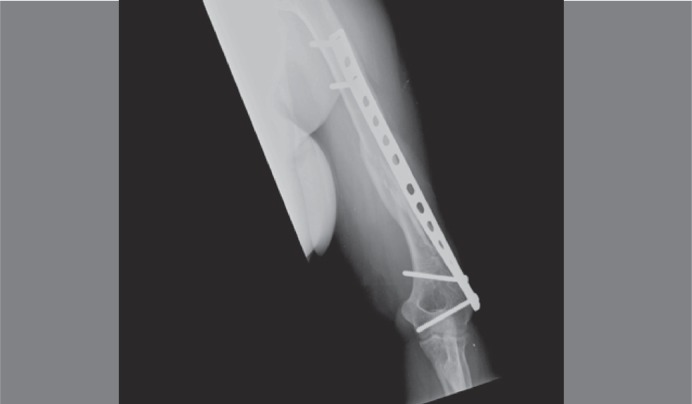
Postoperative X-Ray (AP) of patient treated with MIPO technique and direct visualization of the radial nerve.

**Figure 5:**
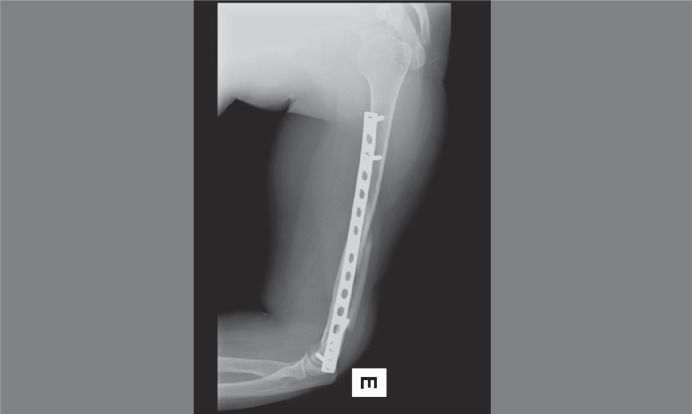
Postoperative X-Ray (Profile) of patient treated with MIPO technique and direct visualization of the radial nerve.

All patients had fracture union, within 4.42 months on average (St.Dev. ± 1.27), as shown in Figures 2-5. The range of motion of the elbow postoperative evolved to functional form. The average flexion-extension was 112.85° (St.Dev. ± 30.93) and the average pronation-supination was 145° (SD ± 22.17), as shown in Table 1. There were no cases of postoperative infection.

The evaluation by the MEPS score showed that four patients had excellent values ​(≥90 points), two were considered good (between 75 and 89 points) and one fair (60 to 74 points). No patient had values ​​below 60 points. The average number of points obtained with the MEPS was 86.42 (St.Dev. ± 15.46). (Table 2)

**Table 2:** Assessment of range of motion and fracture consolidation.

Patient	Time for consolidation (months)	Elbow flexion (degrees)	Elbow extension (degrees)	Forearm pronation (degrees)	Forearm supination (degrees)	MEPS
1	5	120	0	75	80	100
2	4	140	0	75	80	100
3	4	120	0	75	75	80
4	3	140	0	75	80	100
5	4	120	0	75	75	90
6	4	110	10	75	80	75
7	7	90	40	75	20	60
Mean/St. Dev	4.42(±1.27)	120(±17.32)	7.14(±14.96)	75(±0)	70(±22.17)	86.42(±15.46)

## DISCUSSION

Despite the high frequency of fractures of the humeral shaft, around 3-5% of all fractures,^1^^,^ 2 the specific fracture of the distal third is rarer, accounting for about 0.6 to 1%^7^. We also found a low prevalence in our six years study of seven patients.

The mean age of patients included corroborates the data reported in the literature,^9^^,^ 16 as well as the higher prevalence of patients of the masculine gender.^3^^,^ 7 ^-^ 9 ^,^ 16 ^,^ 17

The treatment of fractures of the humeral shaft is mostly performed on the conservative manner.^9^ However, some fractures require surgical approach. Recently, surgical treatment with the use of relative stability through the MIPO technique was recognized by its reproducibility and high rates of bone consolidation.^3^^-^ 9 The original MIPO technique for the treatment of fractures of the distal humerus does not routinely performs the exploration of the radial nerve.^12^

The risk of radial nerve injury during surgical treatment using the MIPO technique is real and should not be underestimated, mainly due to its proximity to the plate^13^ and the possibility of its interposition during reduction.^10^^,^ 13

Due to the high risk of injury to the radial nerve in fractures of the distal humerus, we performed a modification to the original technique. An accessory pathways was performed, which allowed the identification of the nerve and objectively exclude its interposition in the fracture and/or plate after fixation. This modification of the technique was described by Livani *et al*.^7 ^as an option for performing MIPO plate in patients who had, prior to surgery, radial nerve injury. We applied this modification in patients without radial nerve injury prior to surgery.

In our study, there was a higher rate of neuropraxia of the radial nerve, even when comparing to the literature, for fractures treated through the MIPO technique.^16^ However, most studies do no present clear distinction in the degree or type of neurological injury (neuropraxia, substantial injury) and the total time for the return of function. Some studies do not consider neuropraxia, since the final evaluation showed full neurological recovery.^7^^,^ 8

When we compare the index of radial nerve neuropraxia in our work with studies that evaluated it after osteosynthesis of fractures treated with absolute stability, the incidence of neurological impairment was similar.^9^ Therefore, the hypothesis is that neuropraxia occurred due to manipulation of nerve during the course of the accessory pathway. We found no cases of nerve interposition in the fracture focus. However, despite the high rate of neurological impairment, neuropraxia was treated with complete motor and sensory recovery after a maximum of seven months of follow-up. Therefore, the realization of the accessory pathway and identification of the radial nerve were beneficial to objectively exclude nerve interposition in the fracture or between the plate and the bone.

Regarding the consolidation of fractures it was observed in all patients that it was accomplished, as well as demonstrated in the literature.^4^^,^ 7 ^,^ 8 ^,^ 16 ^,17 ^We believe that this is due to the use of minimally invasive technique without aggression to the fracture or to the soft parts.^3^

The findings regarding the range of motion showed no prejudice to the use of accessory pathway for both flexion-extension of the elbow and for pronation-supination of the forearm, as well as those described using the conventional MIPO technique.^3^^,^ 7 ^,^ 8 ^,^ 17 ^-^ 20 All patients evolved to a functional range of motion of the elbow after surgical treatment.

Regarding MEPS, six patients (85.71%) had excellent or good results, in accordance with data found in literature.^18^^-^ 20 Only one patient (14.28%) had regular scoring (60 points), we believe that this is due to the fact that the patient has not made adequate postoperative rehabilitation.

## CONCLUSION

Surgical treatment of fractures of the distal third of the humeral shaft, without previous radial nerve injuries, with the MIPO technique associated to exploration of the radial nerve had a high rate of fracture healing and good evolution of motion and function of the elbow. Despite the transient neuropraxia, the modification of the technique allowed excluding the interposition of this structure on the fracture focus and/or plate.
